# Thrombophlebitis hiding under a KILT – case report on 40 years long-term follow-up of neonatal renal vein thrombosis

**DOI:** 10.1186/s12887-019-1567-7

**Published:** 2019-06-06

**Authors:** Stefan Lauener, Anne Bütikofer, Sandra Eigenheer, Robert Escher

**Affiliations:** 1 Department of Medicine, Spital Emmental, Burgdorf, Switzerland; 2 Department of Radiology, Spital Emmental, Burgdorf, Switzerland

**Keywords:** Neonatal renal vein thrombosis, Renal atresia, Atresia inferior vena cava, Thrombosis, Thrombophlebitis, Long-term anticoagulation, KILT

## Abstract

**Background:**

Neonatal renal vein thrombosis is a recognised cause of renal and inferior caval vein atresia (IVCA). However, the long-term impact of the condition is underrecognized with a high burden of morbidity for the patient, especially in adulthood. IVCA has been shown to be an independent risk factor for deep venous thrombosis (DVT) with a high risk of recurrence. The acronym KILT for kidney and inferior vena cava anomaly with leg thrombosis summarizes the pathological situation.

**Case presentation:**

We present the case of a 40-year-old patient with pain in the right lower limb resulting from acute thrombophlebitis. No risk factors could be identified. His history was remarkable with two episodes of deep venous thrombosis first of the left, then the right leg 22 years earlier; at that time also, no risk factor was identified. Because of the idiopathic character of that thrombosis, the patient remained on long-term anticoagulation with phenprocoumon. The present thrombophlebitis occurred while the INR was not therapeutic in the preceding weeks. A CT with contrast showed atresia of the inferior vena cava and of the right kidney, and presence of numerous collaterals. A thorough medical history revealed a renal vein thrombosis as a neonate. Anticoagulation was intensified, and stent placement became necessary after a further 2 years.

**Discussion and conclusions:**

KILT syndrome is a rare but underrecognized condition. Complications may arise in young adulthood only, and it is of prime importance to instruct parents of the pediatric patient of the possible consequences of renal vein thrombosis and to assure guidance from the treating physicians throughout adulthood. Diagnosis of IVCA is by CT with contrast or by MRI, and lifelong anticoagulation may be necessary. Since the KILT syndrome is widely underdiagnosed, we challenge the clinicians to keep it in mind when confronted with thrombophlebitis or thrombosis of the young, male and with no other identifiable risk factors for deep vein thrombosis.

## Background

Neonatal renal vein thrombosis is a rare but well-recognised complication in the first postnatal month and characterised by the triad of gross hematuria, flank mass (unilateral or bilateral enlargement of kidneys) and thrombocytopenia [1–3]. Up-to-date there are no evidence-based recommendations for treatment of renal vein thrombosis. Although anticoagulation therapy seems to improve the outcome, development of renal atrophy is still a common finding [2, 3], with complications such as hypertension and chronic kidney disease [1–3]. In a large review covering the medical literature from 1992 to 2006, 271 cases of neonatal renal vein thrombosis were identified, and in 70.6% of the cases, the involved kidney became atrophic after the thrombosis [4]. In this context, little has been reported on subsequent vascular pathologies such as thrombus extension in the inferior vena cava (IVC), secondary bypass circulation leading to anatomic variants, varicose changes including recanalization of the umbilical vein and higher risk for deep venous thrombosis [1, 2]. However, thrombus extension from the renal veins to the IVC has been clearly documented [3–5], and an association between neonatal thrombosis and atresia of the IVC has been strongly suggested [6–8]. Moreover, atresia of the IVC has been identified as an underrecognized risk factor for deep-vein thrombosis of the legs [9, 10], and IVC anomalies were found in 16.2% of iliac vein thrombosis [11]. In a survey of an institutional cohort, ten out of 18 patients (56%), aged 12–18 years, suffered from subsequent post-thrombotic syndrome [12]. Finally, the acronym KILT for kidney and IVC anomalies with leg thrombosis has been proposed for the association of kidney atrophy, IVC atresia and thrombosis in the legs [13].

The clinical signs of IVC atresia are usually unspecific such as lower back pain or abdominal pain. Sometimes other organ related symptoms can occur due to malformation of organs that develop in the same embryonic period. Most often, though, patients are asymptomatic [14]. Varices of the leg and / or abdominal wall [7] and deep venous thrombosis can be the first symptoms in later years. Because the initial thrombotic event occurs in the neonatal period, and further thrombotic complications due to IVC atresia mostly arise in young adulthood only [10], the phenomenon is little recognised and described, and doctors might be unaware of the risk of the long-term evolution. This condition can be missed if not specifically and intentionally looked for.

Here we present a case of a confirmed renal vein thrombosis as a neonate and the occurring complications and treatment during a follow-up of over 40 years.

## Case presentation

A 40-year-old patient was sent to our emergency department by his family doctor. He described pain in and a swelling of his right leg, and no fever. During the days before he experienced thoracic pain and difficulties in breathing. There was no history of longer immobilisation, travelling, former surgery or trauma to his leg. He was a smoker. His only medication was phenprocoumon, and the INR had been subtherapeutic (< 2.0) on the two last measurements during the last weeks.

Anticoagulation was first started at age of 18 when a thrombosis of the left leg had occurred. Sonography at that time showed thrombosis from the popliteal to the iliac vein. There was no further imaging study performed at the time, and a thrombophilia screen including measurements of protein S and C, antithrombin and APC resistance was normal. Ten months later, 2 months after cessation of oral anticoagulation, he suffered from a thrombosis of the right leg in the V. iliaca communis, and durable anticoagulation with phenprocoumon was reinstalled. No further investigations were performed at that time.

On examination there was redness and hyperthermia of the right thigh as well as tenderness on palpation along the saphenous and inguinal veins. There were significant varices of both legs and the abdominal wall. The auscultation of the heart and lungs showed no pathological findings. The vital signs were normal as well as the electrocardiogram. The blood sample showed a foreknown renal insufficiency with a moderately elevated creatinine of 132 μmol/l [59–104 μmol/l], an elevated c-reactive protein of 18.2 mg/l [< 5.1 mg/l], and an INR in the therapeutic range.

**Fig. 1:**
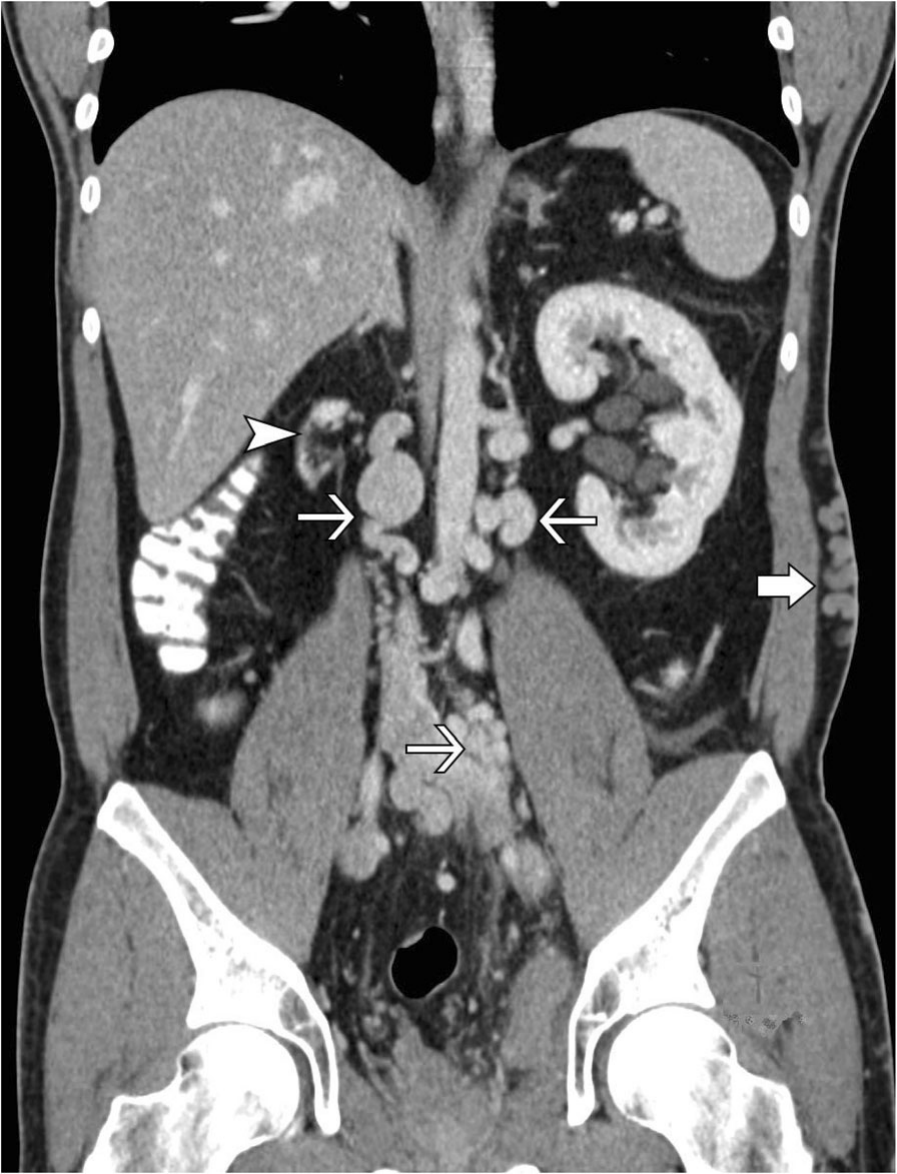
Coronary abdominal CT Scan. CT shows shrunken right kidney (arrowhead), extensive venous collateral system (thin arrows) and subcutaneous collateral vessels (thick arrow)

**Fig. 2:**
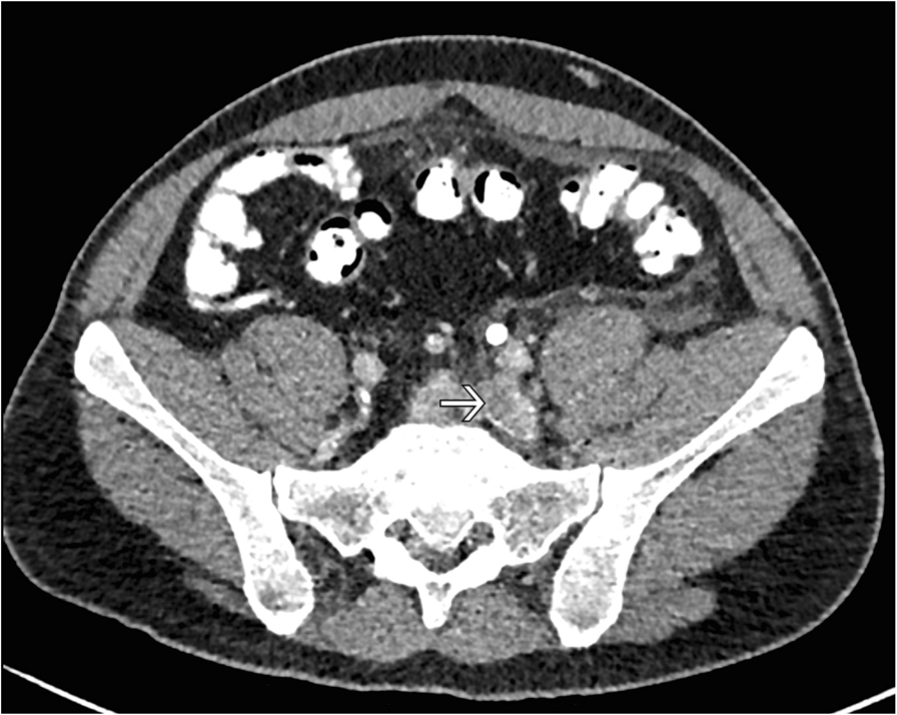
Axial abdominal CT Scan. CT shows left iliac vein thrombosis (arrow)

**Fig. 3:**
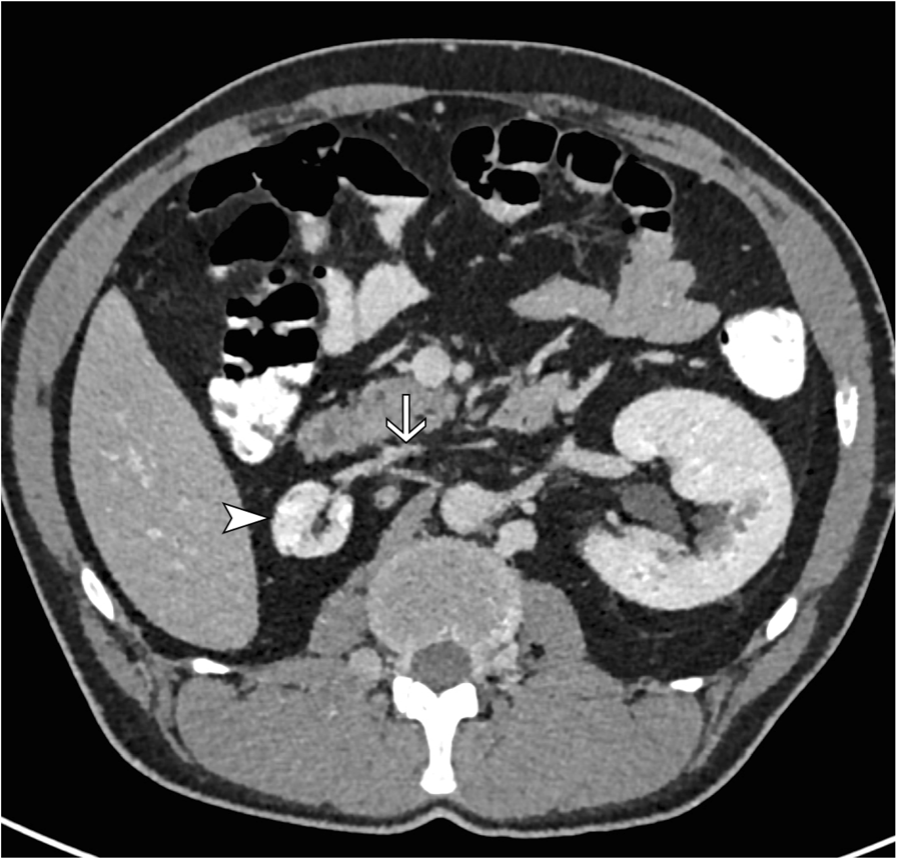
Abdominal CT Scan. CT shows shrunken right kidney (arrowhead) and IVC atresia (arrow)

Duplex sonography of the right limb showed an epifascial thrombophlebitis that barely reached the crossing veins without deep venous thrombosis. A pulmonary embolism was ruled out by a CT-Angiography of the chest. The additional computer tomography of the abdomen and pelvis revealed an abnormal inferior caval vein which was fed just by the hepatic veins. The blood of the extremities and the kidneys was drained over a varicose venous plexus equivalent to the thoracic venae azygos and hemiazygos. In the sense of a bypass circulation the umbilical vein was recanalized, and varicose veins of the abdominal wall were apparent (Fig. 1). The superficial femoral vein on the left side was filled by a probably old thrombus which reached the varicose network in the pelvis (Fig. 2). The right kidney was atrophic (Figs. 1, 3). We diagnosed an epifascial thrombophlebitis of the right leg with missing lower IVC and atrophic right kidney. The history of idiopathic deep vein thrombosis and the present thrombophlebitis could now be explained by the atresia of the inferior caval vein. It has been suggested that this anatomical situation contributes to a prothrombotic situation due to stasis of the blood for lack of sufficient drainage through the azygos and hemiazygos veins [14].

Workup of medical documents during hospitalisation revealed that about 8 years earlier he had been referred to an angiologist where a postthrombotic syndrome of both legs had been diagnosed. Furthermore, an extended medical history including interviews with family members and his treating physician revealed that he suffered from a renal vein thrombosis as a neonate with a consequently shrunken right kidney and impaired renal function (KDIGO Grade 2). The first available written mention of renal size and function was at age 18 when the kidney was described as shrunken and barely visible in ultrasound. Renal function was normal until the age of 35. A functional scintigraphy of the left kidney at the age of 40 revealed a function of 48% which stands for an intact left kidney, the right kidney showing no function at all.

Because of the anatomical abnormality with recurrent deep vein thrombosis we targeted to reach a higher therapeutic INR of 2.5–3.5. Also, to optimize dose adjustments to reach the target INR, the patient successfully started INR self-monitoring. Additionally, he wore strong elastic stockings to prevent swelling, and the patient was instructed to regularly check for ulcers or injuries of the skin.

In the following years, despite a careful anticoagulation, the patient developed more and more a sensation of heavy legs and pressure in the legs. There were signs of postthrombotic vein insufficiency with oedema of the legs, and progressive varicosis of the epifascial abdominal veins representing the cavocaval shunt. After introduction in our country of rivaroxaban as an alternative to the coumarins, the patient opted for this treatment. There had been no other thrombotic events in the meantime. However, at age 42, placement of a vascular cava stent was performed, because of increasing discomfort due to postthrombotic syndrome. This first intervention consisted of endovascular reconstruction of chronic occlusion of the IVC and bilateral pelvic veins (6 Stents). The goal was to improve flow in above mentioned veins and reduce pressure in and extent of collaterals. The patient experienced an immediate reduction of edema, tiredness in the legs, varicosis of abdominal wall as well as in the legs. He could walk longer distances and was overall more satisfied with his quality of life. The medication was changed from phenprocoumon to low molecular weight heparin, then to apixaban. The second elective intervention after 1 month had the goal to further improve blood flow by intravascular ultrasound support to prolong one stent into the suprarenal IVC. After 4 months, however, in-stent thrombosis occurred leading to the third intervention with thrombectomy, PowerPuls® thrombolysis (Actilyse® 10 mg), stent-in-stent reconstruction due to subacute stent thrombosis of cava and bilateral pelvic stents (stents: Sinus XL 22 × 80 mm and Sinus XL 22 × 100 mm in ICV, Sinus XL Flex 14 × 80 mm in right vena iliaca communis and Sinus XL Flex in left vena iliaca communis). After this intervention the medication was changed back from apixaban to coumarin, but added on with clopidogrel 75 mg. A new in-stent thrombosis 1 year later caused the fourth intervention. This time the lysis was performed via catheter over 15 h a day, 2 days in a row, followed by revascularization of the IVC in-stent stenosis by percutaneous transluminal angioplasty (PTA, 20 mm) and placing a stent (Venovo Stent), as well as PTA (14 mm) of the stents in the left pelvic veins. Postoperatively, the medication administered was enoxaparin according to weight (2 × 100 mg daily) and clopidogrel 75 mg daily.

The patient, now 43 years old, is reluctant to restart oral anticoagulation for fear of a recurrent in-stent occlusion, which occurred while under treatment with an oral anticoagulant. In his opinion the parenteral application is safer.

## Discussion and conclusions

This case is remarkable in many aspects. First, the discovery of the IVC anomaly was fortuitous and the computed tomographies were prompted by dyspnoea of the patient and suspicion of pulmonary embolism; secondly, it highlights a more general unawareness of this condition, since haematologists diagnosed a normal laboratory thrombophilia work-up and angiologists a postthrombotic syndrome in both legs, and no abdominal imaging study was performed; thirdly, the case demonstrates the outcome after renal vein thrombosis in the neonate and no long-term anticoagulation at that time; and, lastly, the long-term follow-up of over 40 years in this case is unique, and we did not find a similar case in the literature.

Malformations of the IVC are rare and occur in 0.3–0.5% in healthy people, and it has been shown that in about 0.0005–1% in the general population and 5% of idiopathic thrombosis under age 30 atresia of the IVC (IVCA) is responsible for the thrombosis [9, 10]. As in the present case where a first DVT was diagnosed at 18 years, patients with deep venous thrombosis (DVT) and IVCA are significantly younger (25 ± 6 years) than those without (53 ± 19 years) [15], and bilateral DVT is common [10].

Very little is known about the etiology of IVCA. Two mechanisms are considered responsible for this malformation: embryonic dysontogenesis between 6th–8th week of gestation [16, 17] or an intrauterine or perinatal thrombosis [3–8]. Here, a link between the renal vein thrombosis as a neonate and the IVCA is suggested, further supporting findings from various previous reports [3–8]. It is not surprising that the etiology of IVCA in general is difficult to establish, as IVCA mostly remains asymptomatic until DVT occurs and only rarely leads to various unspecific symptoms such as low back or abdominal pain mainly after intense physical activity, or mostly bilateral varicosis or leg edema [16].

We present a unique long follow-up. Strikingly, despite repeated involvement of physicians, the diagnosis of IVCA remained undetected until advanced adulthood despite the history of renal vein thrombosis as a neonate. Clearly, the awareness of the renal vein thrombosis went missing in the mind of the patient and his parents when transition from pediatric to adult medicine occurred. It stresses the importance of documentation of the neonatal event and of the information of patient, parents and doctors at the beginning as well as during follow-up. As DVT of the legs mostly is diagnosed in young adulthood [15], the information in the literature of pediatric antecedents is scarce [8–11, 14–16]. Here, after a long follow-up, the gap was finally closed.

In 2002, the acronym of KILT syndrome for Kidney and IVC abnormalities with Leg Thrombosis was proposed [13]. Since then several publications referred to this syndrome although it still is an unknown entity to most [18–24]. We present a review of the literature of case reports that fit the acronym of KILT (Table 1). The cases in those reports reflect the young age at diagnosis of DVT (mean 29 years, range 11–54 years). In 7 and 9 case reports including the present case, the right and left kidney was atrophic, respectively. In contrast, in a review on IVCA and DVT, the right kidney was involved slightly more frequently (4.9 and 2.4% for right and left kidney, respectively) [29]. Commonalities in the cases are DVT as the presenting symptom, mostly conservative medical therapy and overall a short follow-up only. For detection of IVCA, CT with contrast or MRI are considered the most accurate modalities, as ultrasound is limited by habitus, bowel gas and because the abdominal vessels cannot be compressed [14, 16, 30]. Also, indefinite anticoagulant therapy as well as wearing of medical compression stockings is advocated [12, 16, 20]. In selected cases of acute DVT in the presence of IVCA, endovascular approaches such as catheter-directed thrombolysis and stenting have been successfully applied [16, 30–32]. Surgery is reserved for cases with severe symptoms [32–36]. Finally, in case of a correct and uninterrupted anticoagulation, the long-term prognosis concerning life expectancy is good [16]. However, there is too limited data to allow a stronger prognostic statement, and the risk for a significant burden of morbidity is high. As in our case, morbidity is likely to increase with age, and, therefore, careful lifelong management is mandatory.

**Table 1:** Reported cases in the literature of DVT, IVCA and atrophic kidney compatible with KILT-syndrome

Author, year of publishing [Ref.]	Sex/ Age	Presentation	Affected kidney	Imaging	Laboratory workup	Therapy	Follow up duration, outcome
Glerup 1994 [25]	M/18	DVT	Right	Cavography, US	NH, TRO	AC	Not reported
Salgado 1998 [26]	M/49	Recurrent DVT, failure to advance catheter	Right	US, Venography, MRI, CT	TRO	AC	Not reported
Timmers 1999 [27]	M/37	DVT, mediastinal mass in chest X-ray, no precipitating factors	Right	US, Chest X-ray, CT, MRI	TRO	UFH, OAC, ES	No recurrence, time unknown
Tsuji 2001 [28]	M/21	DVT	Right	US, CT	TRO	UFH, Urokinase, Warfarin, Aspirin, ES	Not reported
Chee 2001 [10]	F/26	DVT, no precipitating factors	Left	US, CT	TRO	AC lifelong	22 months, no recurrence
Van Veen 2002 [13]	F/16	Bilateral DVT, no precipitating factors	Left	CT	NH	Not reported	Not reported
Gayer 2003 [17]	M/46	Recurrent unilateral DVT	Right	CT	Not reported	Not reported	Not reported
Iqbal 2008 [22]	M/54	Abdominal pain, swelling over right flank, DVT	Left	US, CT	Not reported	LMWH, warfarin	Not reported
Lawless 2012 [23]	M/50	intracranial hemorrhage, DVT, failure to advance catheter	Left	US, MRI	Not reported	Attempted IVC Filter; no AC due to hemorrhage	Not reported
Bami 2015 [20]	M/14	Left leg pain, DVT, no precipitating factors	Left	CT	NH	LMWH then switch to warfarin	Follow up by hematologists
Duicu 2016 [21]	M/12	Abdominal pain, acute thrombosis of renal vein, no precipitating factors	Right	CT; Follow up MRI	TRO	LMWH, warfarin plus antiplatelet, ES	2½ years, OAC, ES
	F/12	Right lower extremity pain, DVT, no precipitating factors,	Left	Angio CT	TRO	LMWH, antiplatelet, higher dose LMWH alone, warfarin	3 months, OAC, ES
Fung 2017 [18]	M/41	Left loin pain, low grade fever, DVT	Left	US, CT	Not reported	LMWH	Follow up by hematologists, duration unknown
Singh 2017 [24]	F/28	Pelvic pain, menorrhagia	Left	US, CT	Not reported	Not reported	Not reported
Pomeranz 2018 [19]	F/11	Left leg pain, limping, low grade fever, DVT	Left	MR venogram	TRO	LMWH, switch to warfarin	3 months
Sagban 2015 [29]^a^	M:F/ 3.2:1	DVT (right sided RR 1.7; both sided RR 2.0)	^a^	US, CT or MRI plus veno-graphy in some cases		AC, when feasible combined with surgery	Not reported

**Abbreviations**: *DVT* deep vein thrombosis, *IVCA* inferior vena cava atresia, *AC* anticoagulation, *ES* elastic stockings, *f* female, *m* male, *NH* normal hemostasis, *OAC* oral anticoagulant, *RR* relative risk, *TRO* thrombophilia ruled out, *UFH* unfractioned heparin, *LMWH* low molecular weight heparin, *US* ultrasound, *CT* computed tomography, *MRI* magnetic resonance imaging

^a^Analysis of 41 patients and literature review of 123 cases with IVCA: hypoplasia/aplasia of left and right kidney in 2.4 and 4.9%, respectively; no case specific details are given

Of note, in DVT with IVCA, pulmonary embolism due to deep vein thrombosis in the legs is unlikely due to the anatomic situation [14, 16]; exceptions are rare [12, 37–39]. In our case, no pulmonary embolism was found, and dyspnoea resolved spontaneously. One might hypothesize that the dyspnoea was due to reduced venous drainage, leading to reduced heart preload. Lastly, an increased prevalence of hereditary coagulation abnormalities has been suggested [29], but a thorough laboratory thrombophilia workup in the present case was negative.

KILT syndrome is a rare phenomenon and conclusions are based upon the available, limited literature as well as upon pathophysiological considerations. It is suggested that in the so-called idiopathic thrombosis in the young patient < 30 years with no thrombophilia or apparent risk factors (immobilisation, contraceptives, trauma), a vascular malformation of the pelvic/central venous system should be considered as well, especially if increased physical exertion precedes DVT [16, 40]. CT with contrast and MRI are the imaging modalities of choice. Long term anticoagulation is recommended, and data on the new oral anticoagulants is limited. Most importantly and as shown by the present case is a thorough information of the patient and the parents at the time of renal vein thrombosis to ensure a lifelong correct management of possible complications, and, when DVT occurs in early adulthood, a thorough workup of the previous medical history targeting at early childhood. The only cost it takes is time, and the benefit may be prevention of further complications by lifelong anticoagulation. Further studies are necessary to provide a broader and more conclusive basis for medical workup and management of patients with KILT syndrome or secondary thrombosis due to IVC atresia.

## Data Availability

Data sharing is not applicable as no datasets were generated. Further details on patient data are available from the corresponding author on reasonable request.

## Abbreviations

CT: Computed tomography
DVT: Deep venous thrombosis
IVC: Inferior vena cava
IVCA: Inferior vena cava atresia
KDIGO: Kidney Disease Improving Global Outcomes
KILT: Kidney and inferior vena cava anomaly with leg thrombosis
LMWH: Low molecular weight heparin
MRI: Magnetic resonance imaging

## References

[1] Moudgil A (2014). Renal venous thrombosis in neonates. Curr Pediatr Rev.

[2] Bidadi B, Nageswara Rao AA, Kaur D, Khan SP, Rodriguez V (2016). Neonatal renal vein thrombosis: role of anticoagulation and thrombolysis--an institutional review. Pediatr Hematol Oncol.

[3] Marks SD, Massicotte MP, Steele BT, Matsell DG, Filler G, Shah PS, Perlman M, Rosenblum ND, Shah VS (2005). Neonatal renal venous thrombosis: clinical outcomes and prevalence of prothrombotic disorders. J Pediatr.

[4] Lau KK, Stoffman JM, Williams S, McCusker P, Brandao L, Patel S, Chan AK, Canadian Pediatric Thrombosis, Hemostasis Network (2007). Neonatal renal vein thrombosis: review of the English-language literature between 1992 and 2006. Pediatrics..

[5] McDonald P, Tarar R, Gilday D, Reilly BJ (1974). Some radiologic observations in renal vein thrombosis. Am J Roentgenol Radium Therapy, Nucl Med.

[6] Ramanathan T, Hughes TM, Richardson AJ (2001). Perinatal inferior vena cava thrombosis and absence of the infrarenal inferior vena cava. J Vasc Surg.

[7] Alicioglu B, Kaplan M, Ege T (2009). Absence of infrarenal inferior vena cava is not a congenital abnormality. Bratisl Lek Listy.

[8] Rogers A, Moloney MA, O'Donnell DH, Sheehan S, Brophy DP (2010). Deep venous thrombosis in a patient with atresia of the infrarenal inferior vena cava. J Vasc Interv Radiol.

[9] Ruggeri M, Tosetto A, Castaman G, Rodeghiero F (2001). Congenital absence of the inferior vena cava: a rare risk factor for idiopathic deep-vein thrombosis. Lancet.

[10] Chee YL, Culligan DJ, Watson HG (2001). Inferior vena cava malformation as a risk factor for deep venous thrombosis in the young. Br J Haematol.

[11] MJ G-F, Forner MJ, Flor-Lorente B, Soler J, Campos S (2006). Inferior vena cava malformations and deep venous thrombosis. Rev Esp Cardiol.

[12] Tarango Cristina, Kumar Riten, Patel Manish, Blackmore Anne, Warren Patrick, Palumbo Joseph S. (2017). Inferior vena cava atresia predisposing to acute lower extremity deep vein thrombosis in children: A descriptive dual-center study. Pediatric Blood & Cancer.

[13] Van Veen J, Hampton KK, Makris M (2002). KILT syndrome?. Br J Haematol.

[14] Paddock M, Robson N (2014). The curious case of the disappearing IVC: a case report and review of the aetiology of inferior vena cava agenesis. J Radiol Case Rep.

[15] Obernosterer A, Aschauer M, Schnedl W, Lipp RW (2002). Anomalies of the inferior vena cava in patients with iliac venous thrombosis. Ann Intern Med.

[16] Lambert M, Marboeuf P, Midulla M, Trillot N, Beregi JP, Mounier-Vehier C, Hatron PY, Jude B (2010). Inferior vena cava agenesis and deep vein thrombosis: 10 patients and review of the literature. Vasc Med.

[17] Gayer G, Zissin R, Strauss S, Hertz M (2003). IVC anomalies and right renal aplasia detected on CT: a possible link?. Abdom Imaging.

[18] Fung JK, Yeung VH, Chu SK, Man CW (2017). KILT (kidney and IVC abnormalities with leg Thrombosis) syndrome in a 41-years-old man with loin pain and fever. Urol Case Rep.

[19] Pomeranz CB, Cullen DL, Bellah RD (2018). Deep venous thrombosis in a child with inferior vena cava and renal anomalies: KILT syndrome. Pediatr Radiol.

[20] Bami Sakshi, Vazquez Yarelis, Chorny Valeriy, Goldfisher Rachelle, Amodio John (2015). Deep Venous Thrombosis of the Leg, Associated with Agenesis of the Infrarenal Inferior Vena Cava and Hypoplastic Left Kidney (KILT Syndrome) in a 14-Year-Old Child. Case Reports in Pediatrics.

[21] Duicu C, Bucur G, Simu I, Tripon F, Marginean O (2016). Deep venous thrombosis associated with inferior vena cava abnormalities and hypoplastic kidney in siblings. Acta Medica Marisiensis.

[22] Iqbal J, Nagaraju E (2008). Congenital absence of inferior vena cava and thrombosis: a case report. J Med Case Rep.

[23] Lawless RA, Dangleben DA (2012). Caval agenesis with a hypoplastic left kidney in a patient with trauma on warfarin for deep vein thrombosis. Vasc Endovasc Surg.

[24] Singh SN, Bhatt TC (2017). Inferior vena cava agenesis: a rare cause of pelvic congestion syndrome. J Clin Diagn Res.

[25] Glerup H, Therkildsen HA (1994). Deep venous thrombosis as a complication of a congenital abnormality of the inferior vena cava. Ugeskr Laeger.

[26] Salgado Ordóñez F, Gavilán Carrasco JC, Bermúdez Recio FJ, Aguilar Cuevas R, Fuentes López T, González Santos P (1998). Absence of the inferior vena cava causing repeated deep venous thrombosis in an adult--a case report. Angiology..

[27] Timmers GJ, Falke TH, Rauwerda JA, Huijgens PC (1999). Deep vein thrombosis as a presenting symptom of congenital interruption of the inferior vena cava. Int J Clin Pract.

[28] Tsuji Y, Inoue T, Murakami H, Hino Y, Matsuda H, Okita Y (2001). Deep vein thrombosis caused by congenial interruption of the inferior vena cava--a case report. Angiology..

[29] Sagban TA, Scharf RE, Wagenhäuser MU, Oberhuber A, Schelzig H, Grabitz K, Duran M (2015). Elevated risk of thrombophilia in agenesis of the vena cava as a factor for deep vein thrombosis. Orphanet J Rare Dis.

[30] Lamparello BM, Erickson CR, Kulthia A, Virparia V, Thet Z (2014). Congenital anomaly of the inferior vena cava and factor V Leiden mutation predisposing to deep vein thrombosis. Vasc Health Risk Manag.

[31] Broholm R, Jørgensen M, Just S, Jensen LP, Bækgaard N (2011). Acute iliofemoral venous thrombosis in patients with atresia of the inferior vena cava can be treated successfully with catheter-directed thrombolysis. J Vasc Interv Radiol.

[32] Harrison B, Hao F, Koney N, McWilliams J, Moriarty JM (2018). Caval Thrombus management: the data, where we are, and how it is done. Tech Vasc Interv Radiol.

[33] Zhou W, Rosenberg W, Lumsden A, Li J (2005). Successful surgical management of pelvic congestion and lower extremity swelling owing to absence of infrarenal inferior vena cava. Vascular..

[34] Sagban TA, Grotemeyer D, Balzer KM, Tekath B, Pillny M, Grabitz K, Sandmann W (2010). Surgical treatment for agenesis of the vena cava: a single-Centre experience in 15 cases. Eur J Vasc Endovasc Surg.

[35] Ali B, Ali Rana M, Langsfeld M, Marek J (2015). A rare cause of claudication treated with IVC reconstruction: a case report. Int J Surg Case Rep.

[36] Dougherty MJ, Calligaro KD, DeLaurentis DA (1996). Congenitally absent inferior vena cava presenting in adulthood with venous stasis and ulceration: a surgically treated case. J Vasc Surg.

[37] Cho BC, Choi HJ, Kang SM, Chang J, Lee SM, Yang DG, Hong YK, Lee DH, Lee YW, Kim SK (2004). Congenital absence of inferior vena cava as a rare cause of pulmonary thromboembolism. Yonsei Med J.

[38] D'Aloia A, Faggiano P, Fiorina C, Vizzardi E, Bontempi L, Grazioli L (2003). Dei Cas L. absence of inferior vena cava as a rare cause of deep venous thrombosis complicated by liver and lung embolism. Int J Cardiol.

[39] Takehara N, Hasebe N, Enomoto S, Takeuchi T, Takahashi F, Ota T, Kawamura Y, Kikuchi K (2005). Multiple and recurrent systemic thrombotic events associated with congenital anomaly of inferior vena cava. J Thromb Thrombolysis.

[40] Koppisetty S, Smith AG, Dhillon RK (2015). Incidental finding of inferior vena cava atresia presenting with deep venous Thrombosis following physical exertion. Case Rep Emerg Med.

